# A compendium of bacterial and archaeal single-cell amplified genomes from oxygen deficient marine waters

**DOI:** 10.1038/s41597-023-02222-y

**Published:** 2023-05-27

**Authors:** Julia Anstett, Alvaro M. Plominsky, Edward F. DeLong, Alyse Kiesser, Klaus Jürgens, Connor Morgan-Lang, Ramunas Stepanauskas, Frank J. Stewart, Osvaldo Ulloa, Tanja Woyke, Rex Malmstrom, Steven J. Hallam

**Affiliations:** 1 Graduate Program in Genome Sciences and Technology, Genome Sciences Centre, University of British Columbia, Vancouver, British Columbia Canada; 2 Department of Microbiology and Immunology, University of British Columbia, Vancouver, British Columbia V6T 1Z3 Canada; 3 Daniel K. Inouye Center for Microbial Oceanography: Research and Education, University of Hawaii, Manoa, Honolulu, HI 96822 USA; 4 School of Engineering, The University of British Columbia, Kelowna, BC Canada; 5 Leibniz Institute for Baltic Sea Research, Warnemünde, Germany; 6 Graduate Program in Bioinformatics, University of British Columbia, Vancouver, BC V6T 1Z4 Canada; 7 Bigelow Laboratory for Ocean Sciences, East Boothbay, ME USA; 8 School of Biological Sciences, Georgia Institute of Technology, Atlanta, GA USA; 9 Center for Microbial Dynamics and Infection, Georgia Institute of Technology, Atlanta, GA USA; 10 Department of Microbiology and Cell Biology, Montana State University, Bozeman, MT USA; 11 Departamento de Oceanografía, Universidad de Concepción, Casilla 160-C, 4070386 Concepción, Chile; 12 Instituto Milenio de Oceanografía, Casilla 1313, 4070386 Concepción, Chile; 13 Department of Energy Joint Genome Institute, Berkeley, CA USA; 14 Environmental Genomics and Systems Biology, Lawrence Berkeley National Laboratory, Berkeley, CA USA; 15 Life Sciences Institute, University of British Columbia, Vancouver, BC V6T 1Z3 Canada; 16 ECOSCOPE Training Program, University of British Columbia, Vancouver, BC V6T 1Z3 Canada; 17 Present Address: Marine Biology Research Division, Scripps Institution of Oceanography, University of California San Diego, La Jolla, CA 92037 USA

**Keywords:** Archaeal genomics, Bacterial genomics

## Abstract

Oxygen-deficient marine waters referred to as oxygen minimum zones (OMZs) or anoxic marine zones (AMZs) are common oceanographic features. They host both cosmopolitan and endemic microorganisms adapted to low oxygen conditions. Microbial metabolic interactions within OMZs and AMZs drive coupled biogeochemical cycles resulting in nitrogen loss and climate active trace gas production and consumption. Global warming is causing oxygen-deficient waters to expand and intensify. Therefore, studies focused on microbial communities inhabiting oxygen-deficient regions are necessary to both monitor and model the impacts of climate change on marine ecosystem functions and services. Here we present a compendium of 5,129 single-cell amplified genomes (SAGs) from marine environments encompassing representative OMZ and AMZ geochemical profiles. Of these, 3,570 SAGs have been sequenced to different levels of completion, providing a strain-resolved perspective on the genomic content and potential metabolic interactions within OMZ and AMZ microbiomes. Hierarchical clustering confirmed that samples from similar oxygen concentrations and geographic regions also had analogous taxonomic compositions, providing a coherent framework for comparative community analysis.

## Background & Summary

**Fig. 1:**
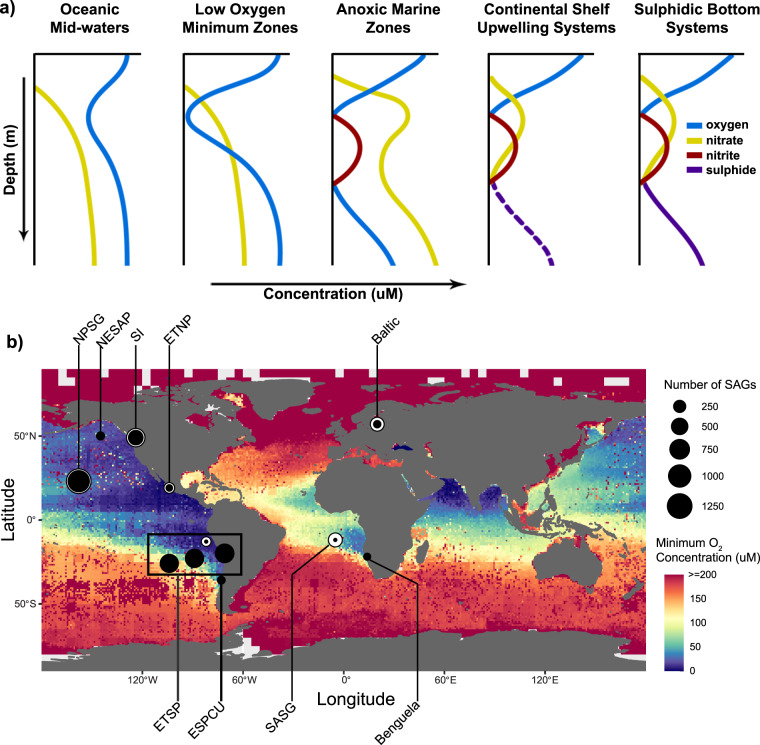
Oxygen minimum zone (OMZ) and anoxic marine zone (AMZ) geochemical profiles and global map of sampling locations. (**a**) The different geochemical profiles of oxygen-deficient marine waters are schematized (modified from Ulloa *et al*., 2012)^4^. Solid lines represent observed data, while the dashed line represent a sporadic accumulation event. (**b**) OMZ and AMZ sampling locations for single-cell amplified genomes (SAGs) are indicated. The total number (white) and sequenced (black) SAGs obtained from each location are denoted with a circle proportional to the corresponding number of samples in the dataset. The Ocean is coloured according to the lowest mean statistical value for the oxygen concentration reported for each 1° and 5° grid in the 2018 annual NOAA World Ocean Atlas^119^, with white grids indicating locations where oxygen concentration data was unavailable. Sampling sites from oceanic midwaters include the North Pacific Subtropical Gyre (NPSG) and the South Atlantic Subtropical Gyre (SASG). Sample sites from low oxygen OMZs include the Northeastern Subarctic Pacific (NESAP). Sample sites from AMZs include the Eastern Tropical North Pacific Gyre (ETNP) and Eastern Tropical South Pacific Gyre (ETSP). Sites from coastal upwelling systems with ephemerally sulfidic bottoms include the Eastern South Pacific Coastal Upwelling (ESPCU) and Benguela coastal upwelling (Benguela). Sampling sites from sulfidic bottom basins include Saanich Inlet (SI) and the Baltic Sea. Geolocalization coordinates and the number of samples for each location are detailed in Table 1.

Oxygen deficient zones are common oceanographic features (Fig. 1) arising when microbial respiratory oxygen demand during breakdown of organic matter exceeds oxygen availability. These waters are operationally defined based on oxygen conditions ranging from dysoxic (20–90 μM), suboxic (1–20 μM), anoxic (less than 1 μM) or anoxic sulfidic (no detectable oxygen)^1,2^. Oceanic midwater oxygen minimum zones (OMZs) such as the North Pacific subtropical gyre present dysoxic conditions capable of supporting anaerobic metabolism through microbial remineralization of sinking particulate organic matter^3^ (Fig. 1a). Low oxygen coastal and open ocean OMZs such as the Northeastern Subarctic Pacific (NESAP) present suboxic conditions encompassing the redox transition for nitrate (NO_3_−) reduction (Fig. 1a). Anoxic marine zones (AMZs) are further differentiated by nitrite (NO_2_−) accumulation with or without sulfide accumulation (sulfidic bottom waters and open ocean or low-oxygen minimum zones (OMZs), respectively)^4–6^. For example, AMZs in the Eastern Tropical North Pacific (ETNP) and Eastern Tropical South Pacific (ETSP) present nanomolar oxygen conditions supporting NO_3_− reduction to NO_2_− and further reduced nitrogen products without hydrogen sulfide (H_2_S) accumulation (Fig. 1a). In contrast, coastal upwelling systems such as Benguela upwelling off the coast of Namibia present episodic shifts in oxygen deficiency, supporting the emergence of transient sulfidic plumes (Fig. 1a). Anoxic sulfidic conditions are also present in coastal fjords, such as the Saanich Inlet (SI), where glacial sills restrict water mass circulation. Sulfidic bottom conditions are also observed in marginal seas, such as the Baltic Sea (Fig. 1a).

Different geochemical profiles within OMZs and AMZs create ecothermodynamic gradients^7^ driving coupled biogeochemical cycling of carbon, nitrogen and sulphur by cosmopolitan and endemic microorganisms adapted for life under low oxygen conditions (reviewed in^2–4,8^). Understanding how these metabolic interactions contribute to nitrogen loss and climate active trace gas production is a critical challenge^9–12^. Global warming exacerbates water column oxygen deficiency through thermal stratification and changes in water mass circulation, resulting in OMZ and AMZ expansion and intensification^13–15^. Other factors, including excessive nutrient inputs (eutrophication), also contribute to coastal and marginal sea oxygen deficiency^15–18^. Efforts to model coupled biogeochemical cycles within OMZs and AMZs using both gene-centric and genome-resolved metagenomic approaches have identified key microbial populations that would benefit directly from availability of improved genome assemblies with increased taxonomic resolution^19,20^.

Cultivation-independent whole genome shotgun sequencing provides direct insights into microbial community structure and function in natural and engineered environments^21–27^. As sequencing technologies improve, it becomes possible to assemble genomes from metagenomes with increasing taxonomic resolution^20^. However, despite an expanding reliance on metagenome-assembled genomes (MAGs), several challenges remain, including resolving population microheterogeneity^28^, incomplete or chimeric genome assemblies (resulting from either assembly or binning), coverage bias, and limited availability of taxonomically characterized reference genomes for cross-validation^29–31^. Advances in fluorescence-activated cell sorting (FACS) and sequencing technologies enable study of uncultivated microorganisms at the individual cell level, providing more accurate taxonomic labels and associated mobile genetic elements (MGEs)^32–38^. Resulting single-cell amplified genomes (SAGs) and MGEs have been used to illuminate coding potential of “microbial dark matter”^39^, provide accurate linkages between taxonomy and function underlying biogeochemical cycles^20,21,30,40^, and to evaluate genome streamlining^41^, fine scale population structure^28,37,42^ and virus-host dynamics^43^. Recent release of the Global Ocean Reference Genomes Tropics, or GORG-Tropics provides a valuable compendium of taxonomically defined SAGs containing >12,000 partial genome sequences from tropical and subtropical euphotic ocean waters^44^. Although a small subset of GORG-Tropics SAGs were collected from ‘oceanic midwater low oxygen’ waters (2,136 of 20,288 sequenced SAGs)^44^, oxygen-deficient marine waters remain conspicuously underrepresented, considering their substantial biogeochemical impact on marine ecosystem functions and services.

**Fig. 2:**
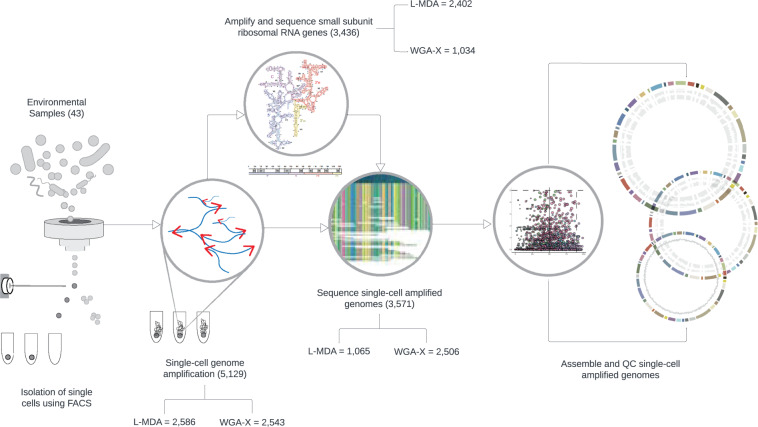
Overview of the workflow for processing and generating microbial Single-cell Amplified Genomes (SAGs). A more detailed scheme is presented in the supplementary information (Supplemental Figure S1-S3) (modified from Rinke *et al*., 2013)^50^.

Here, we present a global compendium of bacterial and archaeal SAGs from OMZs and AMZs. This compendium contains 5,129 taxonomically identified SAGs derived using a combination of targeted and untargeted cell sorting methods, and isolated from environments covering the full range of geochemical profiles associated with extant, oxygen-deficient marine waters^4^ (Fig. 1). Currently, 3,570 of these SAGs have been sequenced, assembled and decontaminated, based on established genomic standards^45^ (Fig. 2, S1a-c). Sequenced and assembled SAGs were processed through the Microbial Genome Annotation Pipeline^46^ for gene prediction and functional annotation, and are available through the Integrated Microbial Genome platform (IMG; https://img.jgi.doe.gov/)^47^ or IMG/ProPortal (https://img.jgi.doe.gov/proportal). The collection of SAG sequences provides an invaluable resource to infer metabolic traits, resolve population structures, and assess spatial and temporal trends of relevant taxonomic lineages within OMZ and AMZ microbiomes.

## Methods

### Sample collection and cryopreservation

Approximately 1–2 mL seawater samples were collected in duplicate or triplicate during various oceanographic cruises within different OMZs and anoxic waters (Fig. 1 and Table 1). Samples were placed in sterile cryovials and amended with one of the following cryoprotectants: glycine betaine (6% [v/v] final concentration^39,48^), glycerol (10% [v/v] final concentration^28,49^), or glycerol-TE buffer^39,50^. Environmental seawater collection was performed using a Niskin-bottle rosette, or a Pump Profiling System for the NBP13-05 cruise (ETSP; *R/V Nathaniel B. Palmer*, July 5–7th, 2013), equipped with a conductivity-temperature-depth profiler, dissolved O_2_ sensor, fluorometer and transmissometer. A modified sample collection protocol was used during the BiG RAPA cruise (ETSP, off the coast of Chile, November 19th 2010, 55 m depth) which was first enriched on-deck selecting for chlorophyll-containing microorganisms^51^. Triplicate samples were passed through a 60 *μ*m size mesh and sorted through an Influx^TM^ (BD Biosciences) flow cytometer system. Approximately 4,000 cells were sorted into 1 mL of sterile glycerol-TE buffer. Sorting was triggered based on the pigment content of particles in the red emission channel (excited by the 488 laser), using forward scattered light as a proxy for particle size. All samples were cryopreserved in liquid nitrogen and then stored at −80 °C, before being processed for single-cell amplified genome generation.

**Table 1:** Number of SAGs generated and sequenced for each sampling location (per depth).

Region	Depth	Month	Year	Site_ID	Lat	Long	Total SAGs	Sequenced SAGs	Reference
Baltic	104.3	Nov	2011	Baltic_104.3_Nov_2011	57.318	20.0511	114	29	This Study
Baltic	109.1	Nov	2011	Baltic_109.1_Nov_2011	57.318	20.0511	110	34	This Study
Baltic	129.1	Nov	2011	Baltic_129.1_Nov_2011	57.318	20.0511	114	20	This Study
Benguela	91	May	2015	Benguela_91_May_2015	−22.3933	14.03183	76	54	^109^
ESPCU_1	80	Mar	2015	ESPCU_1_80_Mar_2015	−36.45	−73	93	64	^51,88,109–113^
ETNP	100	Jun	2013	ETNP_100_Jun_2013	18.9	−104.5	57	19	^51,88,109–113^
ETNP	125	Jun	2013	ETNP_125_Jun_2013	18.9	−104.5	31	15	^51,88,109–113^
ETNP	150	Jun	2013	ETNP_150_Jun_2013	18.9	−104.5	26	6	^51,88,109–113^
ETNP	300	Jun	2013	ETNP_300_Jun_2013	18.9	−104.5	66	25	^51,88,109–113^
ETNP	60	Jun	2013	ETNP_60_2013	18.9	−104.5	5	1	^51,88,109–113^
ETSP_1	20	Nov	2010	ETSP_1_20_Nov_2010	−20.08	−70.8	259	237	^51,88,114,115^
ETSP_1	53	Nov	2010	ETSP_1_53_Nov_2010	−20.083	−70.8	84	45	^51,88,114,115^
ETSP_1	55	Nov	2010	ETSP_1_55_Nov_2010	−20.08	−70.8	323	323	^44,49^
ETSP_2	115	Jul	2013	ETSP_2_115_Jul_2013	−12.998	−82.199	23	11	^51,115^
ETSP_2	250	Jul	2013	ETSP_2_250_Jul_2013	−12.998	−82.199	52	13	^51^
ETSP_2	405	Jul	2013	ETSP_2_405_Jul_2013	−12.998	−82.199	73	13	^51,115^
ETSP_3	112	Nov	2010	ETSP_3_112_Nov_2010	−23.46	−88.77	311	310	^44,49^
ETSP_3	14	Nov	2010	ETSP_3_14_Nov_2010	−23.46	−88.77	328	325	^44,49^
ETSP_4	14	Dec	2010	ETSP_4_14_Dec_2010	−26.25	−103.96	311	307	^44,49^
ETSP_4	180	Dec	2010	ETSP_4_180_Dec_2010	−26.25	−103.96	316	315	^44,49^
NESAP	1000	Jun	2010	NESAP_1000_Jun_2010	50	−145	66	65	This Study
NESAP	3000	Jun	2010	NESAP_3000_Jun_2010	50	−145	35	28	This Study
NPSG	100	Nov	2009	NPSG_100_Nov_2009	22.75	−158	276	272	^44,49^
NPSG	1000	May	2016	NPSG_1000_May_2016	22.75	−158	16	16	^116^
NPSG	125	Dec	2015	NPSG_125_Dec_2015	22.75	−158	53	53	^116^
NPSG	200	Dec	2015	NPSG_200_Dec_2015	22.75	−158	21	21	^116^
NPSG	200	Sep	2009	NPSG_200_Sep_2009	22.75	−158	3	3	^48,50^
NPSG	25	Dec	2015	NPSG_25_Dec_2015	22.75	−158	46	46	^116^
NPSG	25	Sep	2009	NPSG_25_Sep_2009	22.75	−158	147	10	^48,50^
NPSG	3000	Sep	2009	NPSG_3000_Sep_2009	22.75	−158	6	6	^48,50^
NPSG	4000	May	2016	NPSG_4000_May_2016	22.75	−158	38	38	^116^
NPSG	4800	Sep	2009	NPSG_4800_Sep_2009	22.75	−158	10	10	^48,50^
NPSG	5	Aug	2009	NPSG_5_Aug_2009	22.75	−158	302	299	^44,49^
NPSG	500	Dec	2015	NPSG_500_Dec_2015	22.75	−158	33	33	^116^
NPSG	60	Jan	2009	NPSG_60_Jan_2009	22.75	−158	13	13	^42^
NPSG	60	Jul	2009	NPSG_60_Jul_2009	22.75	−158	5	5	^42^
NPSG	750	May	2016	NPSG_750_May_2016	22.75	−158	41	41	^116^
NPSG	770	Sep	2009	NPSG_770_Sep_2009	22.75	−158	245	32	^48,50^
SASG	10	Nov	2007	SASG_10_Nov_2007	−12.4948	−4.99867	89	5	^40,48,117^
SASG	800	Nov	2007	SASG_800_Nov_2007	−12.4948	−4.99867	258	32	^40,48,117^
SI	100	Aug	2011	SI_100_Aug_2011	48.59167	−123.505	248	149	^7,78,118^
SI	150	Aug	2011	SI_150_Aug_2011	48.59167	−123.505	186	116	^7,78,118^
SI	185	Aug	2011	SI_185_Aug_2011	48.59167	−123.505	220	111	^7,78,118^

References shown here point to articles where the SAGs have been studied and/or those offering further sampling and environmental contextual data for these SAGs, as well as cognate metagenomes and/or metatranscriptomes.

### Microbial isolation and Single-cell Amplified Genome (SAG) generation

Samples were thawed and microbial cells sorted at the Bigelow Laboratory for Ocean Sciences’ Single Cell Genomics Center (SCGC) or the Joint Genome Institute (JGI). Samples were passed through a sterile 40 *μ*m size mesh before microorganisms were sorted by either a non-targeted isolation procedure or specific selection for cyanobacteria. For non-target isolation, the microbial particles were labelled with a 5 *μ*M final concentration of the DNA stain SYTO-9 (Thermo Fisher Scientific). Microbial cells were individually sorted using a MoFlo^TM^ (Beckman Coulter) or an InFlux^TM^ (BD Biosciences) flow cytometer system equipped with a 488 nm laser for excitation and a 70 μm nozzle orifice^52^. The gates for the untargeted isolation of microbial cells stained with SYTO-9 were defined based on the green fluorescence of particles as a proxy for nucleic acid content, and side scattered light as a proxy for particle size. For isolation of cyanobacterial cells, gates were defined based on autofluorescence in the red emission channel. An improved discrimination of cyanobacterial cells from detrital particles was performed based on the ratio of green (SYTO-9 DNA label) *versus* red (chlorophyll content) fluorescence. The cytometer was triggered on the side-scatter using the “single-1 drop” mode. All microbial single-cells were sorted into 384-well plates containing 600 nL of 1X TE buffer per well and then stored at −80 °C until further processed. A subset of microbial cells, that generated the SAGs identified with the ‘AAA001’ prefix (part of the SAGs collected at the SASG, 800 m depth), were sorted into ‘prepGEM^TM^ Bacteria reaction mix’ (ZyGEM)^48^. For samples processed in the Bigelow Single Cell Genomics Center, 64 of the 384-wells on each plate were used as negative controls (no droplet deposition), and 3 wells received 10 cells each to serve as positive controls.

The microbial single-cells sorted into TE buffer were lysed as described previously by adding either cold KOH^53^, or 700 nl of a lysis buffer consisting of 0.4 mM KOH, 10 mM EDTA and 100 mM dithiothreitol^52^. Samples were incubated for 10 min at either 4 or 20 °C for samples lysed with cold KOH or lysis buffer, respectively. Microbial cells sorted into ‘prepGEM^TM^ Bacteria reaction mix’ were first lysed following the manufacturer’s instructions and then processed through the cold KOH lysis procedure^48^. The microbial nucleic acids were then whole genome amplified in individual wells through either through traditional Phi29-mediated “Legacy Multiple Displacement Amplification” (L-MDA^39,53^) or using a more thermostable Phi29 polymerase via “Whole Genome Amplification-X” (WGA-X^52^). The products of this procedure are here referred to as SAGs.

### Taxonomic identification of SAGs

While WGA-X generated single-cell genome amplification products were not taxonomically pre-screened, nearly all SAGs processed using L-MDA with the non thermostable polymerase were taxonomically identified by sequencing small subunit ribosomal RNA (SSU or 16 S rRNA) gene amplicons. Both bacterial (27-F: 5′- AGAGTTTGATCMTGGCTCAG -3′^54^, 907-R: 5′- CCGTCAATTCMTTTRAGTTT -3′^55^) and archaeal primers (Arc_344F: 5′- ACGGGGYGCAGCAGGCGCGA -3′^56^, Arch_915R: 5′- GTGCTCCCCCGCCAATTCCT -3′^57^) were used. Real-time PCR and sequencing of the resulting amplicons were performed as previously described^39,52^. Resulting SSU rRNA gene amplicon sequences were queried against the SILVA database v138.1^58^ with blastn, from BLAST + v2.9.0^59^. The top blastn hit (i.e. highest coverage, bit-score, and identity, as well as lowest e-value) was used as the primary taxonomic classification for each pre-screened SAG (Table S1)^60^. Additionally, SSU rRNA gene amplicon sequences were queried against the NCBI-RefSEQ v2021-08-14 database^61^. Top hits were determined using the same criteria described above, and denoted here as the secondary taxonomic assignments of the SAGs (Table S1)^60^. Sequences denoted as “Unclassified” had no significant sequence homology to any of the references within these databases (Table S1)^60^.

Two methods were used to assign taxonomy to the SAGs. Initially, taxonomic assignments for SAGs generated through L-MDA were conducted by extracting SSU rRNA gene sequences directly from the whole genome assemblies, or from the amplicons described above. For all SAGs generated through the WGA-X procedure that were not screened for any phylogenetic marker prior to genome sequencing, a search was conducted to identify SSU rRNA gene sequences > 500 bp within the genome assembly (Supplemental Figure S2). This search was performed through the Integrated Microbial Genomes & Microbiome system (IMG/M, https://img.jgi.doe.gov/m/)based on its gene prediction and annotation pipeline (see below)^45,46^. Additionally, SSU rRNA sequences were recovered from a subset of SAG assemblies with the *Recovering ribosomal RNA gene sequences* workflow with Anvi’o v7.0^62^. These SSU rRNA gene sequences were processed as described above to assign taxonomy (Table S1)^60^. Because 1,281 SAGs did not provide sufficient SSU rRNA gene sequence information (Table S2)^60^, all SAG assemblies were also processed through the Genome Taxonomy Database Tool Kit GTDB-Tk v2.1.0^63–70^ with GTDB R07-R207_v2^71–73^ reference data for multi-locus taxonomic assignment. This allowed for taxonomic identification of SAGs missing SSU rRNA gene sequences, and offered an additional reference compared to those assigned by partial or complete phylogenetic marker sequences. The number of taxonomic assignments that were generated using both methods are detailed in Table S3^60^, with the assignments being available in Table S1^60^.

### Genome sequencing, de novo assemblies and decontamination

SAGs were sequenced as described previously^39,52^, and their reads assembled into contigs using SPAdes v2.2.10 to v3.10.0^74^. Contigs of <2,000 bp were removed from SAG assemblies. Completeness and contamination levels of SAG assemblies were then determined using CheckM v1.2.1^75^. To comply with established genomic standards^45^, assemblies exceeding 5% estimated contamination were run through ProDeGe v2.2 to v2.3^76^ to eliminate the conflicting contigs until there was no improvement in their contamination estimates. The contamination and completeness levels for these SAGs were then re-evaluated using CheckM v1.2.1^75^ and those that still exceeded 5% contamination were manually decontaminated through the Metagenomics Workflow and Refining MAG bin workflows available in Anvi’o v5^62^.

### Manual decontamination of Saanich Inlet SAGs

A total of 14 SAGs exceeded 5% contamination after being processed through the ProDeGe decontamination pipeline^76^ and short-contig trimming (Table S5)^60^. These SAGs were manually decontaminated with Anvi’o v5 using the Metagenomics Workflow and Refining MAG bin workflows^62^. A contig database and the corresponding Hidden Markov Model for each database was generated for each of these SAGs. The taxonomy for each gene was then assigned using the Centrifuge Database^77^. Additional manual curation of these SAGs was carried out using differential coverage of each SAG based on metagenomic reads from Saanich Inlet metagenomes (August 2011 100 m, 150 m, and 2012 100 m, 150 m. Biosamples SAMN05224439, SAMN05224444, SAMN05224441, SAMN05224518, BioProject PRJNA247822)^78^. Raw metagenomic reads were mapped with bwa *v0.7.17-r1188*^79^ and samtools *v1.6-19-g1c03df6*^80^. Anvi profile databases were generated for each SAG by utilizing the contig databases and the read mapping files. Individual contigs were manually removed through the interactive interface based on taxonomic identity, average tetranucleotide identity, and low differential coverage. The new assemblies were exported as fasta files and re-assessed with CheckM.

### SAG quality classification

After CheckM was run on all decontaminated SAG assemblies, the quality of each SAG was determined based on Bower*s et al. 2017*^45^. SAGs that were <50% estimated completeness were considered low quality SAGs. SAGs that had ≥50% estimated completeness and <10% estimated contamination were considered to be at least medium quality. To determine if a SAG was high quality, in addition to having >90% estimated completeness and <5% estimated contamination, SAGs need to have 23 S, 16 S, and 5 S rRNA genes and at least 18 tRNAs present in the final assembly. To identify and quantify the rRNAs and tRNAs, SAGs were passed through Barrnap v0.9 (https://github.com/tseemann/Barrnap)^81^ and tRNA-SE v2.0.11^82^ respectively. Any SAGs having >90% estimated completeness and <5% estimated contamination but missing one or more rRNA genes with at least 18 tRNAs were classified as medium quality. The rRNA and tRNA counts, as well as Quality classifications for each SAG can be found Table S1^60^.

### Genome annotation

All genome assemblies were annotated through the Joint Genome Institute’s IMG platform and annotated using the JGI Microbial Genome Annotation Pipeline^46^. The IMG (https://img.jgi.doe.gov/) or IMG/ProPortal (https://img.jgi.doe.gov/proportal) systems host all final assembled and decontaminated SAG sequences, with gene calls and functional annotations publicly available through these portals. All IMG accession numbers for sequenced SAGs are provided (Table S1)^60^.

### Hierarchical clustering

**Fig. 3:**
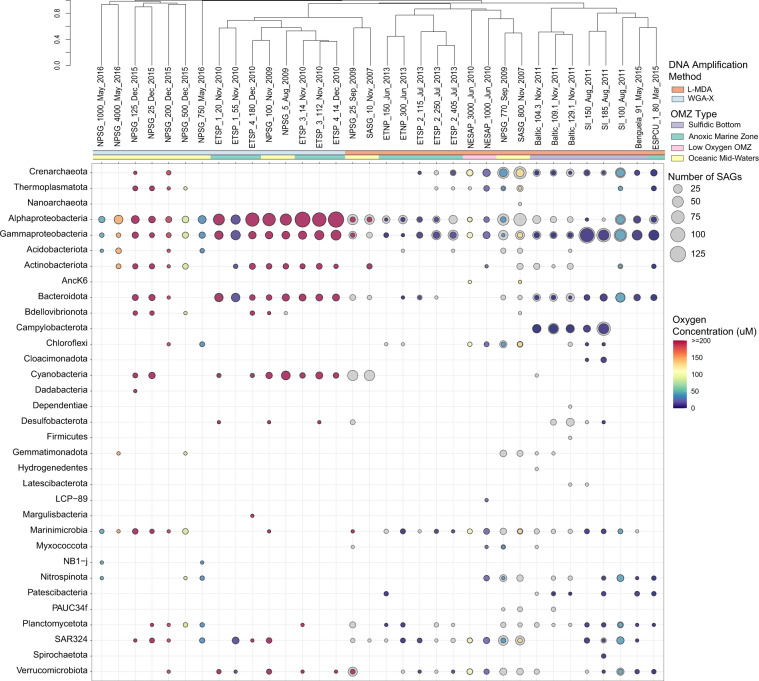
A SAG-based assessment of microbial composition across OMZs. The dot-plot presents the taxonomic designation and proportion of anonymously sorted SAGs sequenced (colored dot) in each taxa at the phylum level and Proteobacteria at the class level from each location. Underlying grey dots represent SAGs collected and taxonomically screened, but not currently sequenced. Taxonomy was determined by SSU rRNA gene amplicon sequences as defined by SILVA v138.1. Dot colour represents environmental oxygen concentrations at time of sampling. Sampling locations were clustered according to the similarity of the SAG taxonomic composition collected at each location. Clustering scale represents the Bray-Curtis dissimilarity among the microbial diversity from each location based on SAG sequence information. Annotation bars denote DNA amplification mentod and OMZ type. Location information is colour encoded as shown for DNA amplification method, OMZ or AMZ type, and oxygen concentration at time of sampling. Sampling location names, on the tips of the dendrogram, are denoted as ‘location_depth (m)_collection month and/or year’. Location acronyms correspond to: Saanich Inlet (SI), Northeastern Subarctic Pacific (NESAP), North Pacific Subtropical Gyre (NPSG), Eastern Tropical North Pacific (ETNP), Eastern Tropical South Pacific (ETSP), Eastern South Pacific Coastal Upwelling (ESPCU), Benguela coastal upwelling (Benguela), South Atlantic Subtropical Gyre (SASG), and the Baltic Sea (Baltic).

The recovered SSU rRNA gene amplicon sequences covering the V4-V5 variable region were clustered at 97% identity using CD-Hit^83–85^, and assigned identifiers based on a representative sequence from each cluster. Based on the taxonomic identity of these representative sequences, the proportion of SAGs associated with each cluster was determined on a per sample basis. These proportions were used to calculate Bray-Curtis Dissimilarity indices using the vegdist() command in the vegan R package v2.5-7^86^. The samples were clustered based on Bray-Curtis dissimilarity, using an average linking method for hierarchical clustering using the hclust command in base R and visualized (Fig. 3).

## Data Records

*File 1*: *Table S1. OMZ SAG biosamples with associated cruise and geolocation metadata. This file contains all Bioproject and Biosample accessions, IMG genome IDs, SRA accessions, Genbank accessions, CheckM outputs, GTDB-tk outputs, and SSU rRNA BLAST results can be found in*: *Table S1_Metadata-template-Bio-Med-SAGdescriptor-OMZ_April_06_2023.xlsx (10.6084/m9.figshare.20481603)*^60^.

*File 2*: *Table S2. Number of SAGs generated with each DNA amplification method, and how many recoverable SSU rRNA gene sequences were recovered from each dataset. Note that there are some samples that had both amplicon and whole genome derived SSU rRNA gene sequences. This information can be found in (10.6084/m9.figshare.20539005)*^60^ *and*: https://github.com/hallamlab/OMZ_SAG_Compendium_Figures/blob/main/Outputs/Table_S2_Summary_Table_WGA_Approach_Mar_21_2023.csv

*File 3***:** *Table S3. Number of SAGs that were assigned a taxonomy with SILVA v138.1 and GTDB-tk v2.1.0 and their summary CheckM % completeness and % contamination estimates. This information can be found in (10.6084/m9.figshare.20539056)*^60^ *and*: https://github.com/hallamlab/OMZ_SAG_Compendium_Figures/blob/main/Outputs/Table_S3_QA_QC_Summary_Mar_21_2023.csv

*File 4*: *Table S4. Primary and secondary contact for each 384 microwell plate that contains the SAGs used in this compendium. This information can be found in Table S4_PI_Contact_Info.xlsx (10.6084/m9.figshare.20483595)*^60^.

*File 5*: *A zip-file compressed tar archive containing the genomic assemblies (10.6084/m9.figshare.20459526)*^60^*.fna – Nucleic acid file in multi-fasta format*

*File 6*: *A zip-file compressed tar archive containing the genomic SSU rRNA gene sequences, and the partial SSU rRNA gene amplicon sequences used for taxonomic assignment can be found in (10.6084/m9.figshare.20537919)*^60^*.fna – Nucleic acid file in multi-fasta format*

*File 7*: *Table S5*. *An xlsx file containing the list of SAGs that underwent manual decontamination from Saanich Inlet, as well as the depth they originated from. The depths were used to select the metagenome reads used for the manual decontamination process. This table can be found in (10.6084/m9.figshare.20538936)*^60^.

## Technical Validation

Early implementation of SAG workflows involved MDA of anonymously sorted single cells in 384-well plate format followed by PCR amplification of selected phylogenetic markers e.g., SSU rRNA gene, to identify SAGs of interest for sequencing^39^. Recent development of WGA-X coupled with low-coverage genome sequencing (LoCoS) provides a more economical workflow to identify hundreds of SAGs per sample without potential PCR bias^52^. Targeted methods of sorting based on spectral properties of cells or substrates have also been applied to SAG selection and sequencing, including cyanobacteria and cells binding to fluorescently labelled substrates, such as cellulose^87–89^. Although the SSU rRNA gene remains one of the most extensively used phylogenetic markers and has well-established and curated databases (e.g. SILVA^58^), multi-locus phylogenetic assignment tools, such as GTDB-Tk^63–70^ generate equally valid results using more information. For this compendium, microbial diversity was assessed using taxonomic labels and abundance information for SAGs sequenced using non-targeted cell-sorting approaches. However, not all SAGs had a match for their SSU rRNA gene taxonomy due to either their amplicon sequences being too short (188 L-MDA SAGs with < 500 bp amplicons; Table S2) or no SSU rRNA gene was recovered from the random genome amplification (*i.e*. 1,093 WGA-X SAGs; Table S2). Thus, an additional taxonomic classifier was run for all SAGs, based on whole-genome assignment using GTDB-Tk^63–70^. Both sets of classifications are in Table S1. Hierarchical clustering (of 3,217 SAGs that contained an assignable V4-V5 SSU rRNA gene amplicon sequence) revealed a higher similarity among those from depths and geographic locations with similar oxygen conditions (Fig. 3), a result consistent with prior observations^2–4,8^. It should also be noted that many of the SAGs amplified with the WGS-X method originated from highly oxygenated samples, which had similar taxonomic compositions and therefore clustered together. Based on this information, the OMZ and AMZ SAG sequences presented here should serve to complement previous SAG collections obtained from (oxygenated) euphotic ocean waters^44,49^.

## Usage Notes

This compendium is intended to fill a critical gap in taxonomically labelled reference genomes from marine oxygen-deficient waters. Included SAG sequences were processed using well-established assembly and decontamination workflows. However, links to the raw data are also available for users interested in using future software versions or implementing alternative workflows. Any approach should aim to discern contaminating sequences associated with FACS (co-sorting two or more cells into a single well, environmental DNA contamination) and WGA (reagent contamination)^90^. It is important to emphasize that SAG sequences often contain MGEs, including plasmids and viruses^43,91–93^. These sequences are typically filtered out during the decontamination process, although differentiating between endogenous chromosomal intervals such as islands or prophage from MGEs requires careful manual curation. Users interested in MGEs are encouraged to work with the raw data or initial assemblies prior to decontamination. Note that genome assembly contamination estimates obtained by CheckM should be handled with caution, as this tool is prone to both over and under estimating completeness and contamination^94^. As described above, recent advances in MDA using WGA-X have led to improved SAG completion and the adoption of LoCoS has obviated the need for SSU rRNA gene amplicon screening to select SAGs of interest for sequencing^52^. The sequences included in this compendium include both older and more contemporary SAG sequencing approaches. The results are integrated by presenting SSU rRNA gene and multi-locus taxonomic assignments based on SILVA, NCBI, and GTDB.

**Fig. 4:**
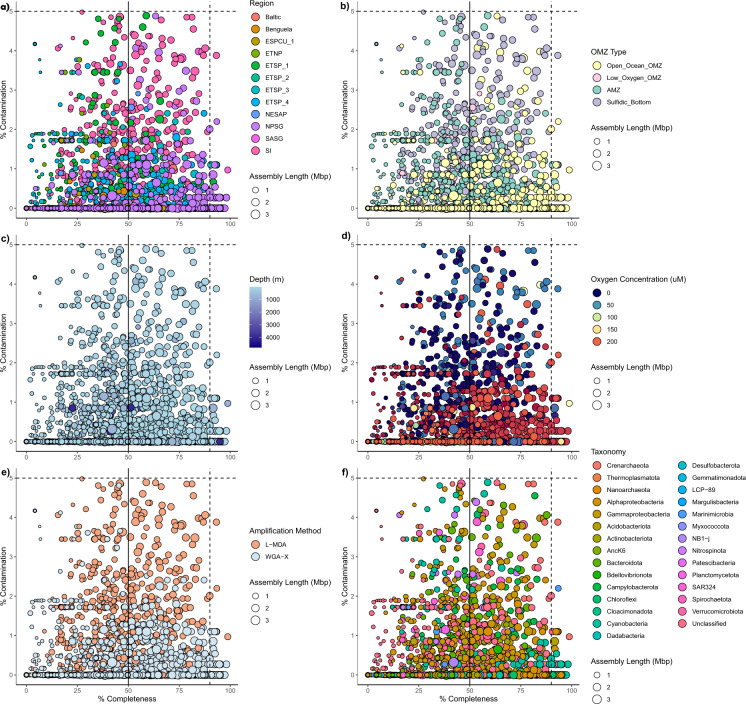
CheckM completeness and contamination estimates of sequenced SAGs for all sequenced SAGs with the point size representing the assembly length in Megabase Pairs (MBP). Of these, the solid line represents the estimated completeness and contamination threshold for medium quality SAGs (> = 50% Completeness, <10% Contamination) and the dashed line represents the threshold for high quality SAGs (>90% Completeness, <5% Contamination)^45^. Plots are coloured based on (**a**) region, (**b**) OMZ ecotype, (**c**) depth, (**d**) environmental oxygen concentration level, (**e**) DNA amplification method, and (**f**) taxonomic group (class level for Proteobacteria, phylum level for other taxa) as defined by SILVA v138.1. Note that SAGs >5% estimated contamination have been excluded from this figure.

Despite improvements introduced with WGA-X^52^, single-cell genomics invariably results in incomplete genome assemblies (Fig. 4, Supplemental Figure S4). This limitation can be overcome in part when multiple SAGs sharing extremely high levels of nucleotide identity are obtained from the same sample. Such closely related sequences can be analysed together, enabling more complete metabolic reconstruction^7,33,42,51^, or used to generate combined assemblies^50,95^. In addition, population-level genomes can be obtained through hybrid assemblies combining SAG sequences and metagenomic sequences^96,97^. In all cases, SAG contigs should be quality filtered to eliminate the presence of contaminating sequences and comply with established genomic standards^45^. All SAG assemblies reported here were thoroughly decontaminated, reaching <5% contamination for all except four SAGs (that only had between 5–10% contamination; Fig. 4, Supplemental Figure S4, Table S2).

Many SAGs included in this compendium have not been sequenced, and the DNA remains in storage. Users are encouraged to identify underrepresented microorganisms from OMZ and AMZ microbiomes based on the provided taxonomic information that can be prioritized for sequencing and shared with the user community. At the same time, we recognize that there are also underrepresented OMZ and AMZ environments not included in this compendium. Sequences from the Black Sea, South China Sea, Arabian Sea and Bay of Bengal, among others, would provide a more robust representation of oxygen-deficient marine waters for use in comparative studies and modelling efforts. Finally, the SAG sequences included in this compendium can be used as taxonomically characterized reference genomes to recruit metagenomic data sets from marine environments, improve pathway prediction methods^29,98–106^ or expand reference packages for gene-centric analysis of functional markers^107^.

## Supplementary information

Supplimentary Figures

## Data Availability

The scripts used to calculate the number of SAGs, the Bray-Curtis Dissimilarity Matrix, conduct the hierarchical cluster, and generate the Figs. 1b, 3, 4, Supplemental Figures S4–S8 written under R version 4.1.3. These scripts utilize the following R packages: tidyverse, egg, vegan, dendexted, sf, rnaturalearth, and rnaturalearthdata will produce the tables and figures presented in this paper. Direct link to relevant software and specifications can be found online at the Hallam Lab Github repository https://github.com/hallamlab/OMZ_SAG_Compendium_Figures. Additional software used, including version numbers, adjustable variables and other parameters include the following: *Trimmomatic 0.35*^108^: *-phred33 LEADING:0 TRAILING:5 SLIDINGWINDOW:4:15 MINLEN:36* *ILLUMINACLIP:Trimmomatic-0.35*^108^: */adapters/TruSeq. 3-PE.fa:2:3:10 LEADING:3 TRAILING:3 SLIDINGWINDOW:4:15 MINLEN:36* *SPAdes 3.0.0-3.10* ^74^ *: --careful--sc--phred-offset 33* *ProDeGe v2.3.0* ^76^ *CheckM v1.2.1*^75^: *checkm lineage_wf --tab_table -x.fna --threads 8 --pplacer_threads 8* *CheckM v1.2.1*^75^: *checkm qa -o 2 --tab_table* *GTDB-Tk v2.1.0*^63–70^: *gtdbtk classify_wf --genome_dir --out_dir -x.fna --cpus 8* *Nucleotide-Nucleotide BLAST 2.9.0*+^59^: *blastn -query -db -outfmt “6 qacc sacc stitle staxid pident bitscore” -max_target_seqs. 1 -num_threads 4 -out* *Nucleotide-Nucleotide BLAST 2.9.0*+^59^: *blastn -query -db -outfmt “6 qacc stitle pident bitscore” -max_target_seqs. 1 -num_threads 4 -out* *Anvi’o v5*^62^: *anvi-gen-contigs-database -f -o -n* *Anvi’o v5*^62^: anvi-run-hmms -c *Anvi’o v5*^62^: anvi-get-sequences-for-gene-calls -c -o *Anvi’o v5*^62^: $CENTRIFUGE_BASE/p + h + v/p + h + v gene-calls.fa -S centrifuge_hits.tsv *Anvi’o v5*^62^: anvi-import-taxonomy-for-genes -c -p *BWA v 0.7.17-r1188*^79^:*bwa index* *BWA v 0.7.17-r1188*^79^:*bwa mem* *Samtools v 1.6-19-g1c03df6 (using htslib 1.6-55-gb065a60)*^80^: samtools view -b F 4 *Samtools v 1.6-19-g1c03df6 (using htslib 1.6-55-gb065a60)*^80^: samtools index file.sorted.bam *Anvi’o v5*^62^: anvi-profile -i -c --min-contig-length 2000 --output.dir --cluster-contigs *Anvi’o v5*^62^: anvi-merge path_to_profile1/PROFILE.db path_to_profile2/PROFILE.db -o --skip-concoct-binning *Anvi’o v5*^62^: anvi-interactive -p *Anvi’o v5*^62^: anvi-summarize -c -p -C *Anvi’o v7*^62^: anvi-gen-contigs-database -f -o *Anvi’o v7*^62^: anvi-run-hmms -c --num-threads 8 *Anvi’o v7*^62^: anvi-get-sequences-for-hmm-hits *barrnap*^81^: barrnap --kingdom bac --threads {threads} --outseq {working_dir}/barrnap/*rRNA.fasta {input.fasta_dir}/$g.fasta > {working_dir}/barrnap/$g.rRNA.gff *barrnap v0.9*^82^: barrnap --kingdom arc --threads {threads} --outseq {working_dir}/barrnap/*rRNA.fasta {input.fasta_dir}/$g.fasta >{working_dir}/barrnap/$g.rRNA.gff *tRNAscan-SE v 2.0.11*^82^: tRNAscan-SE -B -o {working_dir}/trnascan/$g.output.txt -m {working_dir}/trnascan/$g.stats.txt -b {working_dir}/trnascan/$g.bed -j {working_dir}/trnascan/$g.gff -a {working_dir}/trnascan/$g.trna.fasta -l {working_dir}/trnascan/$g.log --thread {threads} {input.fasta_dir}/$g.fasta
