# Supplementary material for: A compendium of bacterial and archaeal single-cell amplified genomes from oxygen deficient marine waters
Source: Sci Data. 2023 May 27;10:332. doi: 10.1038/s41597-023-02222-y (PMC10224968; doi:10.1038/s41597-023-02222-y)
Supplement: Supplementary file 1 — Supplimentary Figures [file 41597_2023_2222_MOESM1_ESM.pdf]

Supplementary Figures

Figure S1. Detailed pre-processing pipeline of water samples of Pre-processing workflow........................ 2
Figure S2. DNA amplification and genomic sequencing workflow. 3
Figure S3. Genome assembly and decontamination workflow. 4
Figure S4. Quality assessment of SAG assemblies.. 6
Figure S5. CheckM completeness and contamination estimates of sequenced SAGs faceted by each sample location 8
Figure S6. CheckM completeness and contamination estimates of sequenced SAGs for each taxonomic group (class level for Proteobacteria, phylum level for other taxa) as defined by SILVA v138.1. 10
Figure S7. CheckM completeness and contamination estimates of sequenced SAGs for each taxonomic group (class level for Proteobacteria, phylum level for other taxa) as defined by SILVA v138.1. 12
Figure S8. CheckM completeness and contamination estimates of sequenced SAGs for each taxonomic group (class level for Proteobacteria, phylum level for other taxa) 14

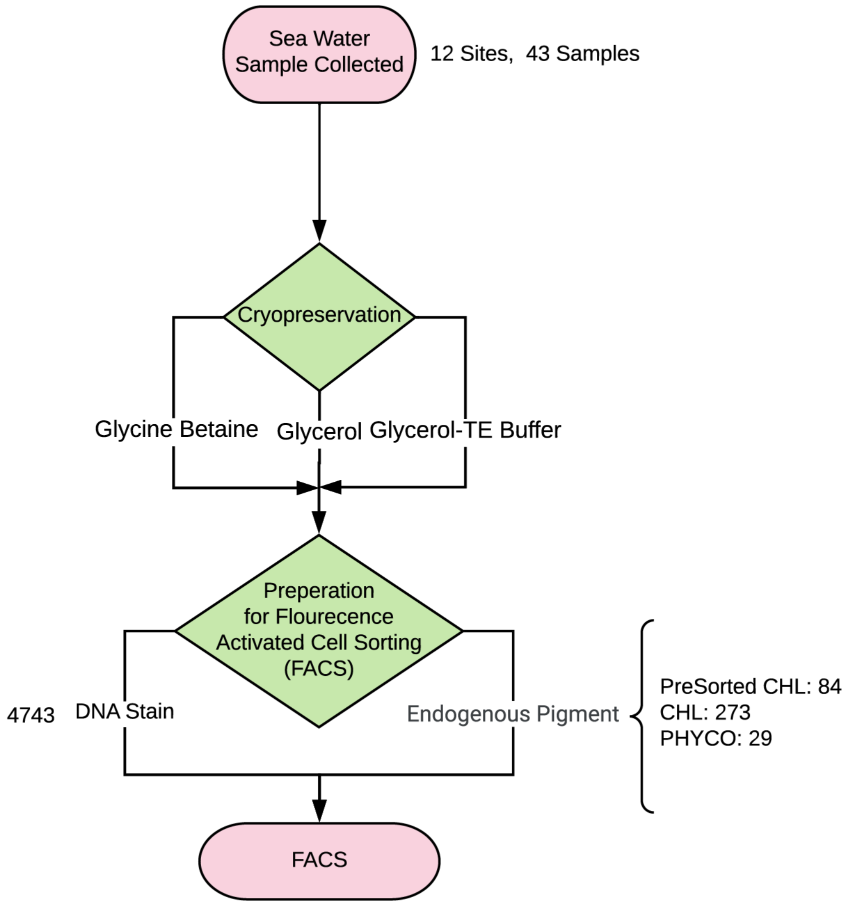

Figure S1. Detailed pre-processing pipeline of water samples of Pre-processing workflow. 4,743 were sorted gating for the fluorescence of a DNA dye that was added to the sample, 84 where SAGs were obtained from a natural sample that was isolated for single-cell amplification by Fluorescence Activated Cell-Sorting (FACS), gating for the fluorescence of a DNA dye that was added to the sample (Presort CHL), 273 SAGs were isolated for single-cell amplification by FACS, gating for the red fluorescence of chlorophyll-containing cells, 29 SAGs were isolated for single-cell amplification by FACS, gating for the orange fluorescence of phycoerythrin-containing cells (PHYCO).

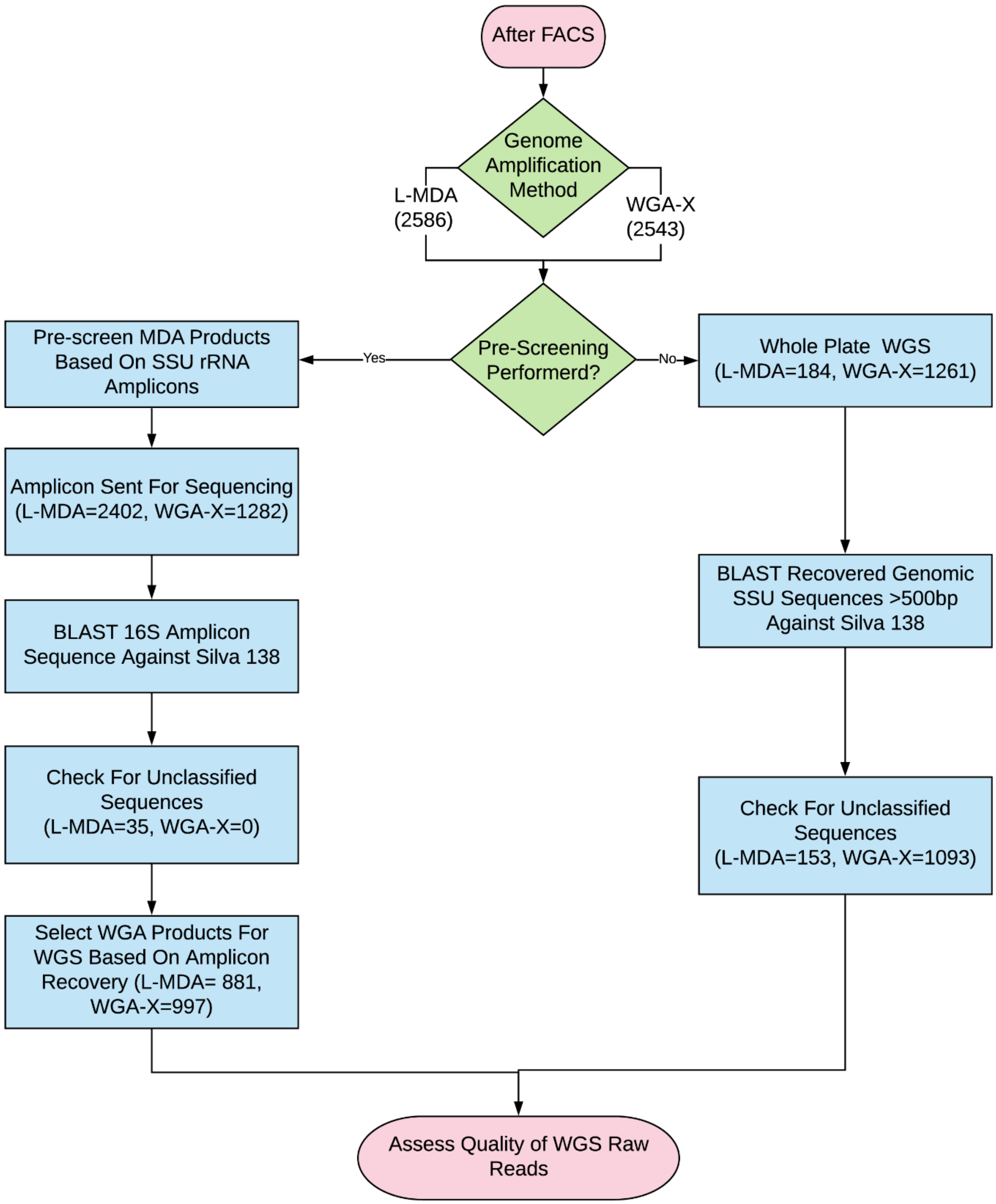

Figure S2. DNA amplification and genomic sequencing workflow.

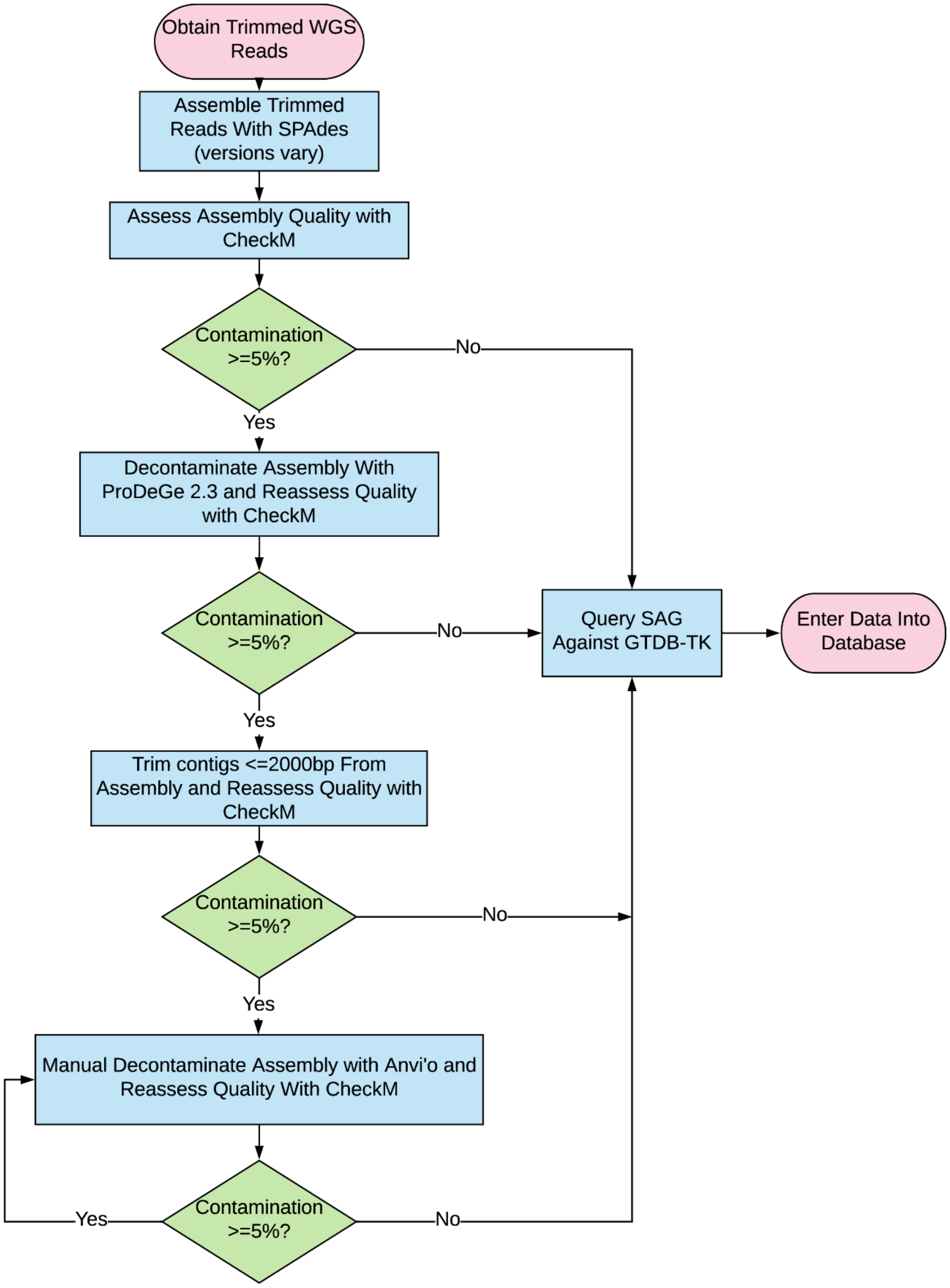

Figure S3. Genome assembly and decontamination workflow.

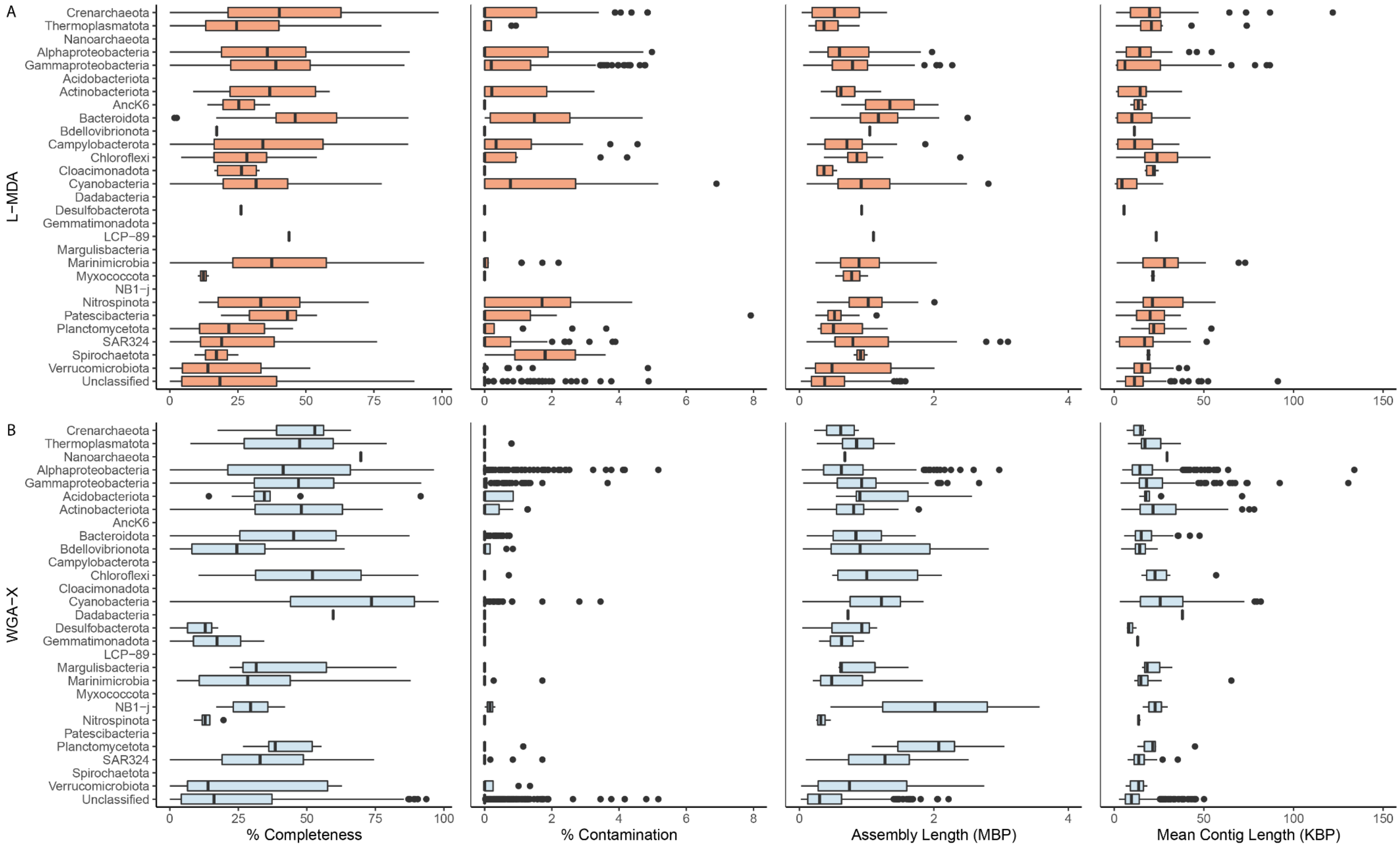

A
L−MDA
B
WGA−X
Crenarchaeota
Thermoplasmatota
Nanoarchaeota
Alphaproteobacteria
Gammaproteobacteria
Acidobacteriota
Actinobacteriota
AncK6
Bacteroidota
Bdellovibrionota
Campylobacterota
Chloroflexi
Cloacimonadota
Cyanobacteria
Dadabacteria
Desulfobacterota
Gemmatimonadota
LCP−89
Margulisbacteria
Marinimicrobia
Myxococcota
NB1−j
Nitrospinota
Patescibacteria
Planctomycetota
SAR324
Spirochaetota
Verrucomicrobiota
Unclassified
% Completeness
% Contamination
Assembly Length (MBP)
Mean Contig Length (KBP)

Figure S4. Quality assessment of SAG assemblies. Box plots present the estimated completeness and contamination levels per CheckM, as well as genome assembly sizes and mean contig lengths for SAGs generated through L-MDA (A) or WGA-X (B).

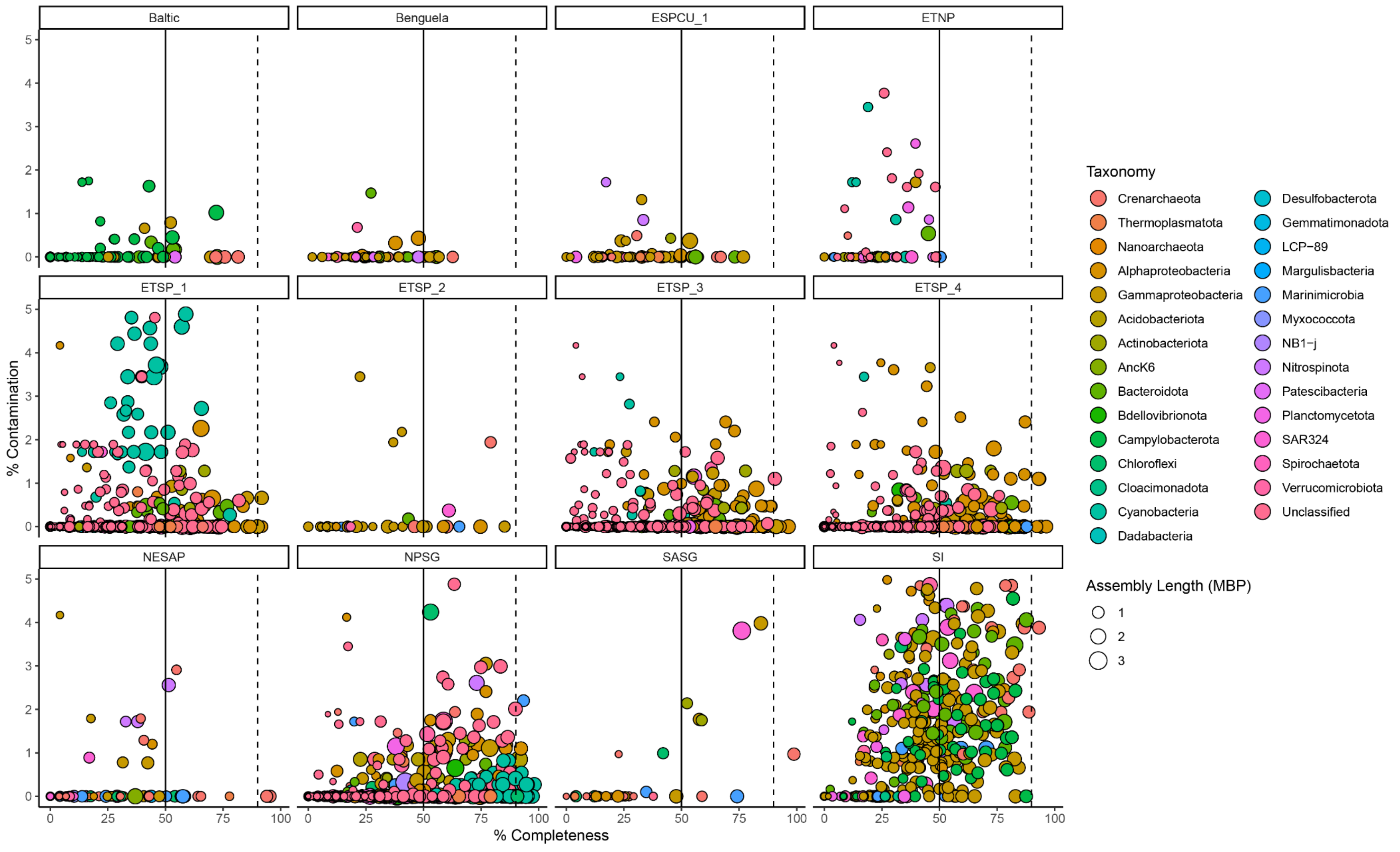
Baltic
Benguela
ESPCU_1
ETNP
ETSP_1
ETSP_2
ETSP_3
ETSP_4
NESAP
NPSG
SASG
SI
% Contamination
% Completeness
Taxonomy
Crenarchaeota
Thermoplasmatota
Nanoarchaeota
Alphaproteobacteria
Gammaproteobacteria
Acidobacteriota
Actinobacteriota
AncK6
Bacteroidota
Bdellovibrionota
Campylobacterota
Chloroflexi
Cloacimonadota
Cyanobacteria
Dadabacteria
Desulfobacterota
Gemmatimonadota
LCP−89
Margulisbacteria
Marinimicrobia
Myxococcota
NB1−j
Nitrospinota
Patescibacteria
Planctomycetota
SAR324
Spirochaetota
Verrucomicrobiota
Unclassified
Assembly Length (MBP)
1
2
3

Figure S5. CheckM completeness and contamination estimates of sequenced SAGs faceted by each sample location. The solid line represents the estimated completeness and contamination threshold for medium quality SAGs and the dashed line represents the threshold for high quality SAGs46. Data points are coloured based on taxonomy (class level for Proteobacteria, phylum level for other taxa) as defined by SILVA v138.1, with the diameter of each point representing assembly length in Megabase Pairs (MBP). Note that SAGs >5% estimated contamination have been excluded from this figure.

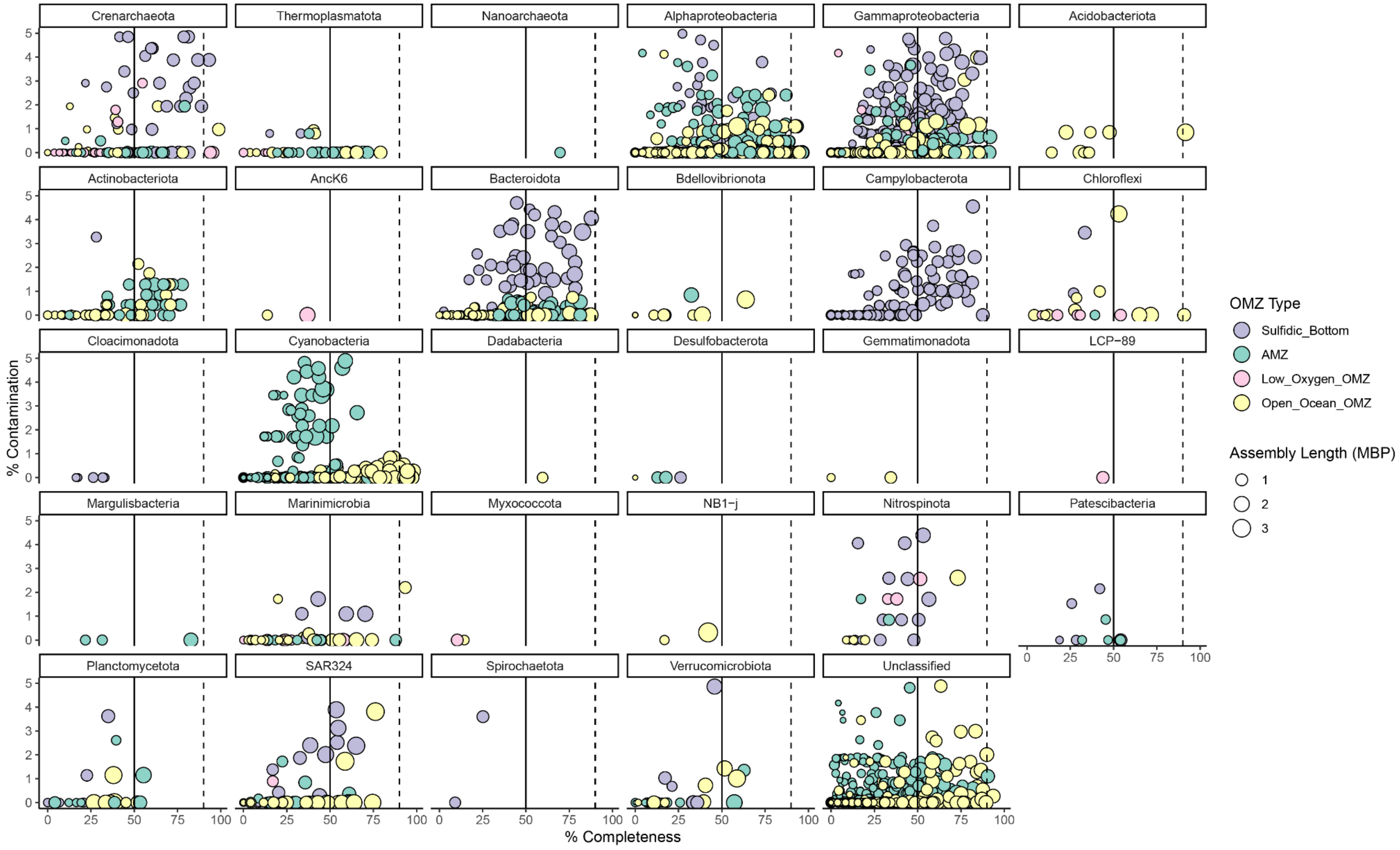
Crenarchaeota
Thermoplasmatota
Nanoarchaeota
Alphaproteobacteria
Gammaproteobacteria
Acidobacteriota
Actinobacteriota
AncK6
Bacteroidota
Bdellovibrionota
Campylobacterota
Chloroflexi
Cloacimonadota
Cyanobacteria
Dadabacteria
Desulfobacterota
Gemmatimonadota
LCP−89
Margulisbacteria
Marinimicrobia
Myxococcota
NB1−j
Nitrospinota
Patescibacteria
Planctomycetota
SAR324
Spirochaetota
Verrucomicrobiota
Unclassified
% Contamination
% Completeness
OMZ Type
Sulfidic_Bottom
AMZ
Low_Oxygen_OMZ
Open_Ocean_OMZ
Assembly Length (MBP)
1
2
3

Figure S6. CheckM completeness and contamination estimates of sequenced SAGs for each taxonomic group (class level for Proteobacteria, phylum level for other taxa) as defined by SILVA v138.1. The solid line represents the estimated completeness and contamination threshold for medium quality SAGs and the dashed line represents the threshold for high quality SAGs46. Samples are coloured based on the OMZ ecotype, with the size of each point representing the length of each assembly in Megabase Pairs (MBP). Note that SAGs >5% estimated contamination have been excluded from this figure.

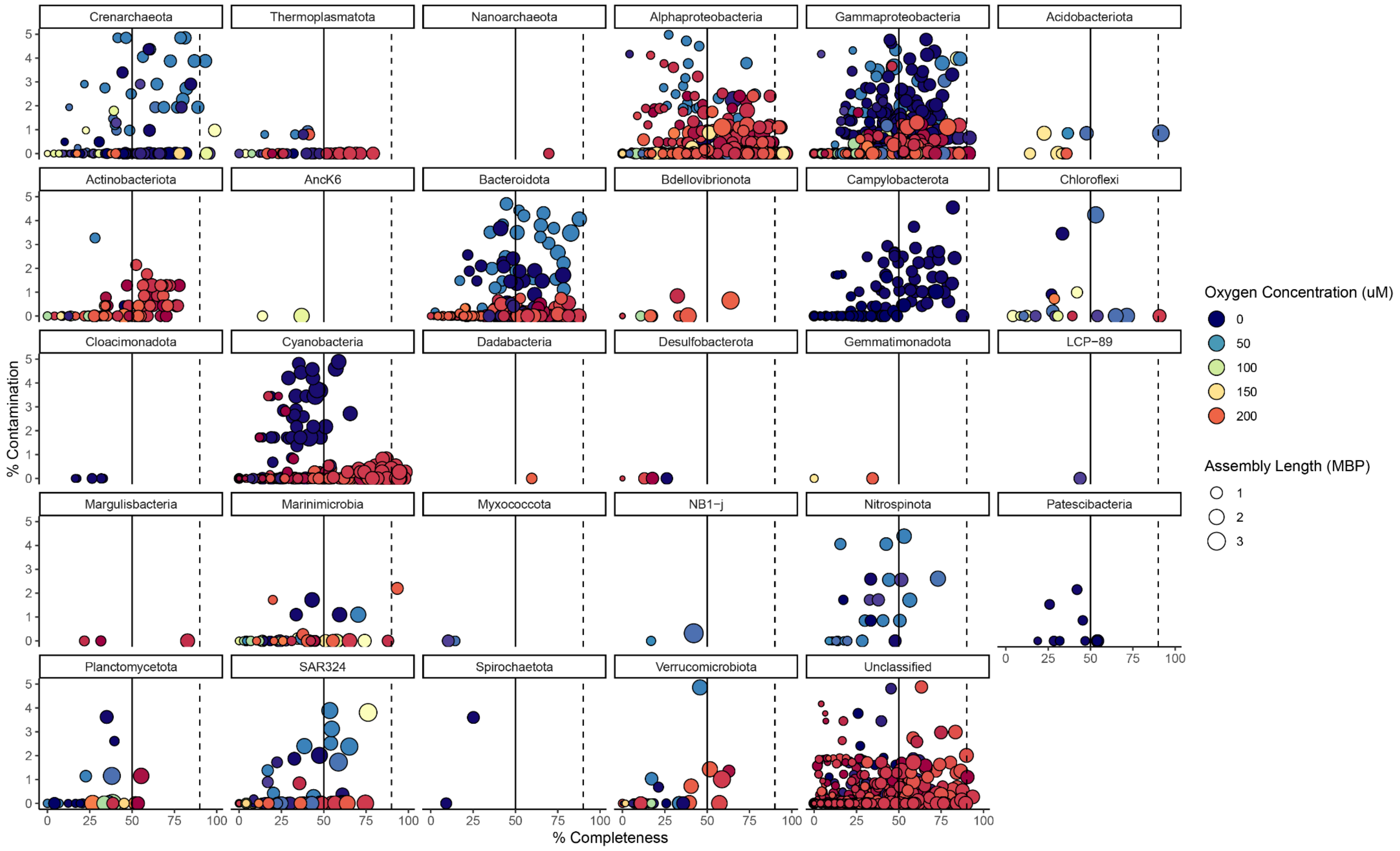

Crenarchaeota
Thermoplasmatota
Nanoarchaeota
Alphaproteobacteria
Gammaproteobacteria
Acidobacteriota
Actinobacteriota
AncK6
Bacteroidota
Bdellovibrionota
Campylobacterota
Chloroflexi
Cloacimonadota
Cyanobacteria
Dadabacteria
Desulfobacterota
Gemmatimonadota
LCP−89
Margulisbacteria
Marinimicrobia
Myxococcota
NB1−j
Nitrospinota
Patescibacteria
Planctomycetota
SAR324
Spirochaetota
Verrucomicrobiota
Unclassified
% Contamination
% Completeness
Oxygen Concentration (uM)
0
50
100
150
200
Assembly Length (MBP)
1
2
3

Figure S7. CheckM completeness and contamination estimates of sequenced SAGs for each taxonomic group (class level for Proteobacteria, phylum level for other taxa) as defined by SILVA v138.1. The solid line represents the estimated completeness and contamination threshold for medium quality SAGs and the dashed line represents the threshold for high quality SAGs46. Samples are coloured based on the environmental oxygen conditions, with the diameter of each point representing assembly length in Megabase Pairs (MBP). Note that SAGs >5% estimated contamination have been excluded from this figure.

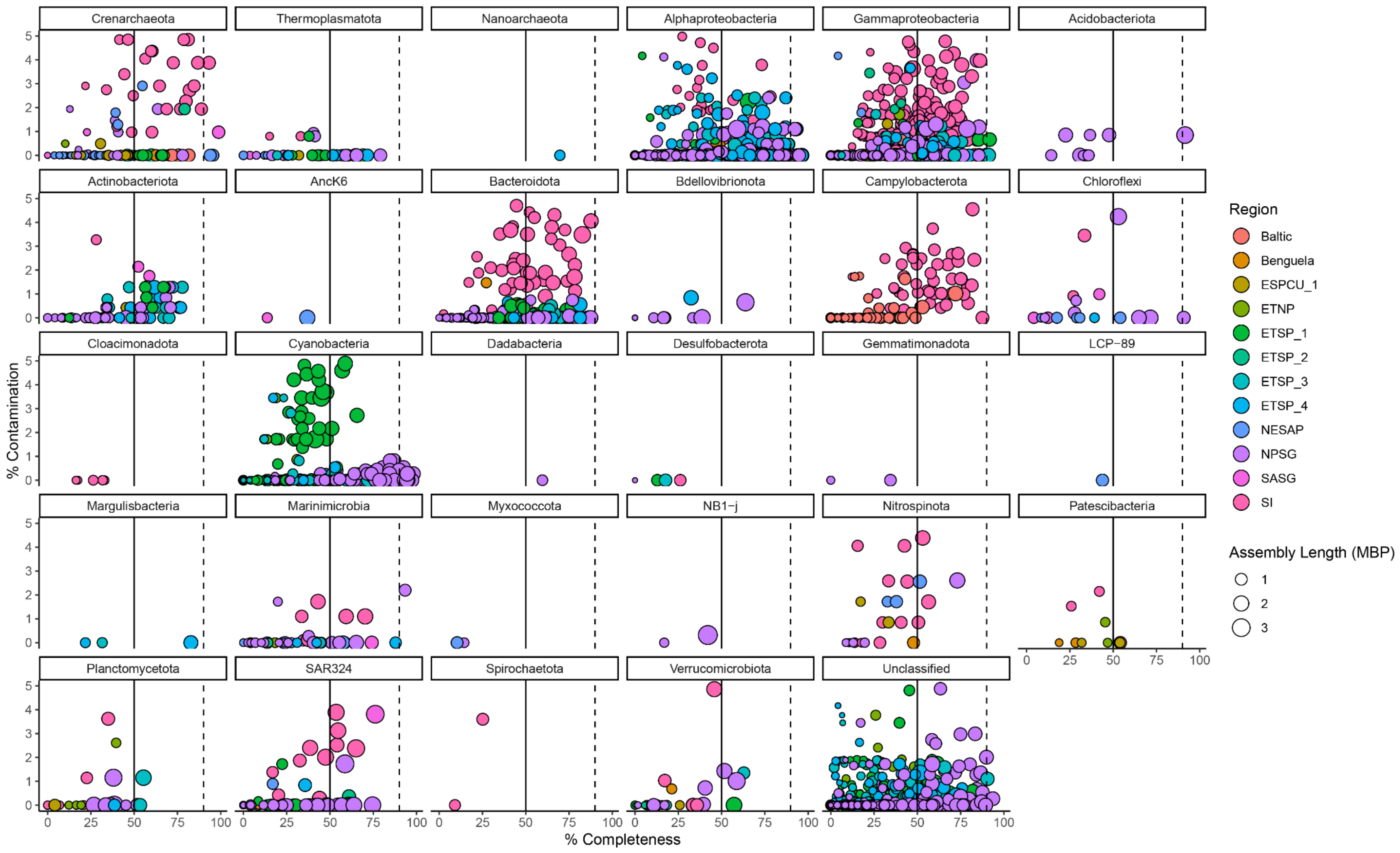

Crenarchaeota
Thermoplasmatota
Nanoarchaeota
Alphaproteobacteria
Gammaproteobacteria
Acidobacteriota
Actinobacteriota
AncK6
Bacteroidota
Bdellovibrionota
Campylobacterota
Chloroflexi
Cloacimonadota
Cyanobacteria
Dadabacteria
Desulfobacterota
Gemmatimonadota
LCP−89
Margulisbacteria
Marinimicrobia
Myxococcota
NB1−j
Nitrospinota
Patescibacteria
Planctomycetota
SAR324
Spirochaetota
Verrucomicrobiota
Unclassified
% Contamination
% Completeness
Region
Baltic
Benguela
ESPCU_1
ETNP
ETSP_1
ETSP_2
ETSP_3
ETSP_4
NESAP
NPSG
SASG
SI
Assembly Length (MBP)
1
2
3

Figure S8. CheckM completeness and contamination estimates of sequenced SAGs for each taxonomic group (class level for Proteobacteria, phylum level for other taxa). The solid line represents the estimated completeness and contamination threshold for medium quality SAGs and the dashed line represents the threshold for high quality SAGs46. Samples are coloured based on the region they originated from, with the diameter of each point representing assembly length in Megabase Pairs (MBP). Note that SAGs >5% estimated contamination have been excluded from this figure.
